# Correction: Evaluation of deacetylase inhibition in metaplastic breast carcinoma using multiple derivations of preclinical models of a new patient-derived tumor

**DOI:** 10.1371/journal.pone.0251106

**Published:** 2021-04-29

**Authors:** Tiffany C. Chang, Margarite D. Matossian, Steven Elliott, Hope E. Burks, Rachel A. Sabol, Deniz A. Ucar, Henri Wathieu, Jovanny Zabaleta, Luis Del Valle, Sukhmani Gill, Elizabeth Martin, Adam I. Riker, Lucio Miele, Bruce A. Bunnell, Matthew E. Burow, Bridgette M. Collins-Burow

The ninth author’s name is spelled incorrectly. The correct name is: Luis Del Valle.

The author’s initials are also indexed incorrectly in PubMed. The correct initials are: Del Valle L.
