# Supplementary figures and images for: Evaluation of deacetylase inhibition in metaplastic breast carcinoma using multiple derivations of preclinical models of a new patient-derived tumor
Source: PLoS One. 2020 Oct 9;15(10):e0226464. doi: 10.1371/journal.pone.0226464 (PMC7546483; doi:10.1371/journal.pone.0226464)

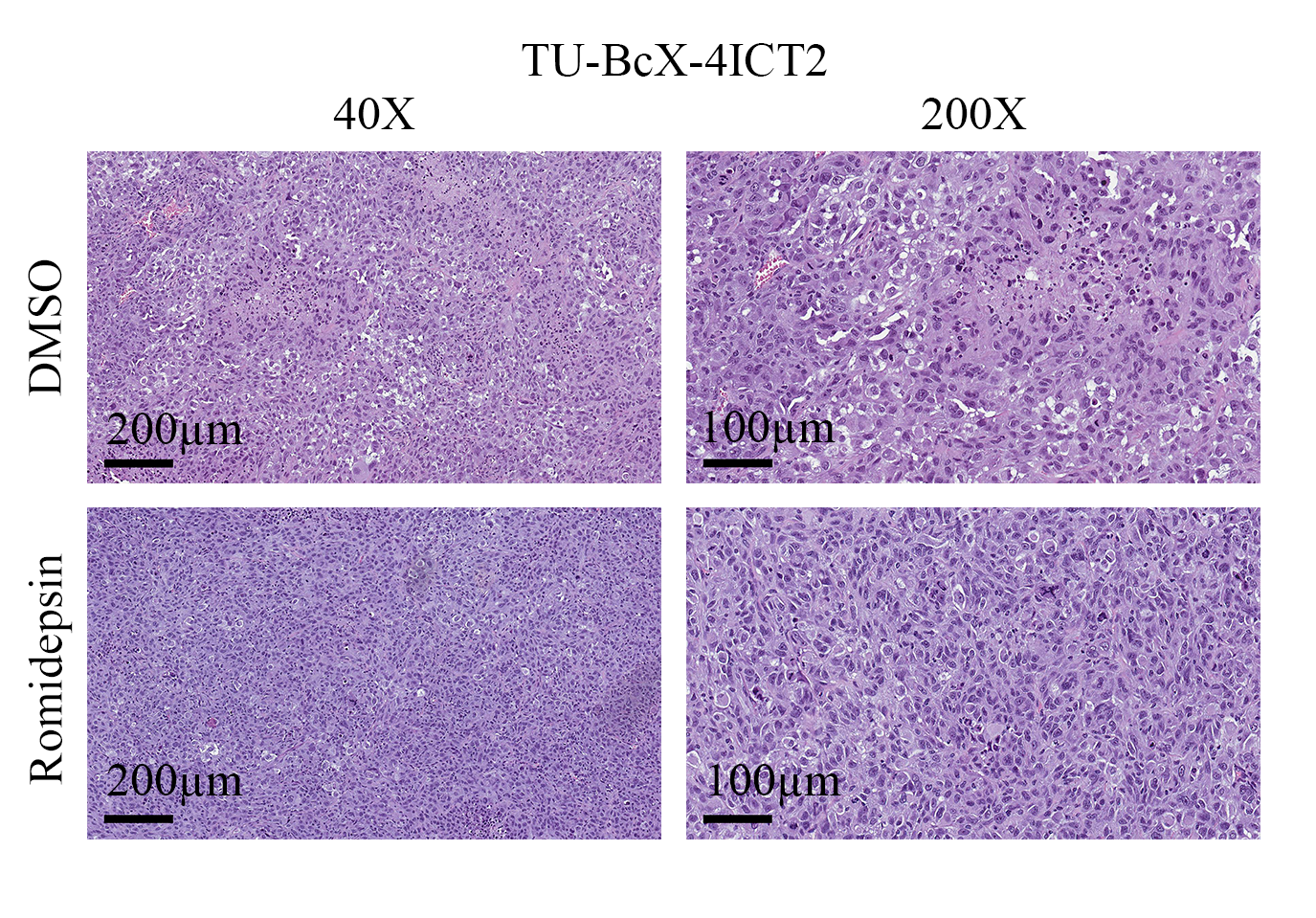

Supplement: S1 Fig — Tumors were excised, formalin-fixed, paraffin-embedded, and H & E-stained to visualize cellular composition and changes in romidepsin-treated tumors compared to DMSO control. Atypical histologic features were seen on both romidepsin and DMSO tumor specimens, including mitotic figures, nuclear pleomorphism, and hyperchromicity. (TIF) [file pone.0226464.s001.tif]

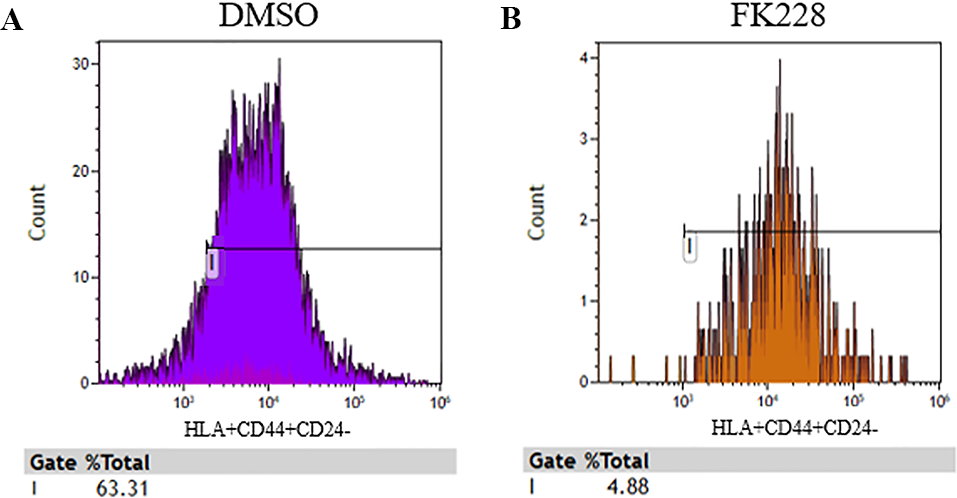

Supplement: S2 Fig — Representative histogram of circulating tumor and stem cell populations, defined as HLA+CD44+CD24-, in mice treated with (A) DMSO and (B) romidepsin were generated in Kaluza Analysis 2.1 Software (Beckman Coulter). (TIF) [file pone.0226464.s002.tif]

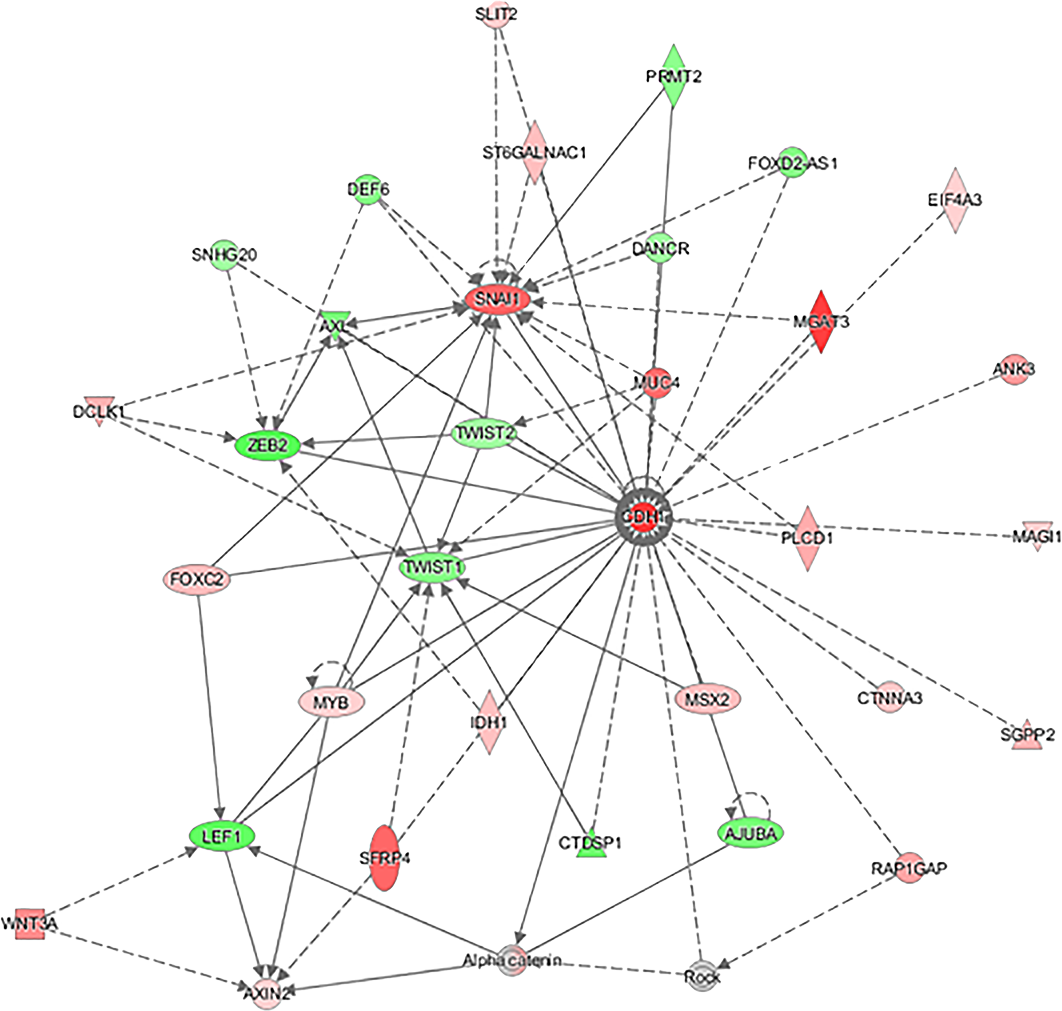

Supplement: S3 Fig — Pathway analyses demonstrate the networks of EMT-related gene changes in romidepsin-treated TU-BcX-4IC cells compared to DMSO control treated cells. Data is shown as Log2 (fold change). Genes highlighted by green represent upregulated genes and downregulated genes are highlighted in red. (TIF) [file pone.0226464.s003.tif]

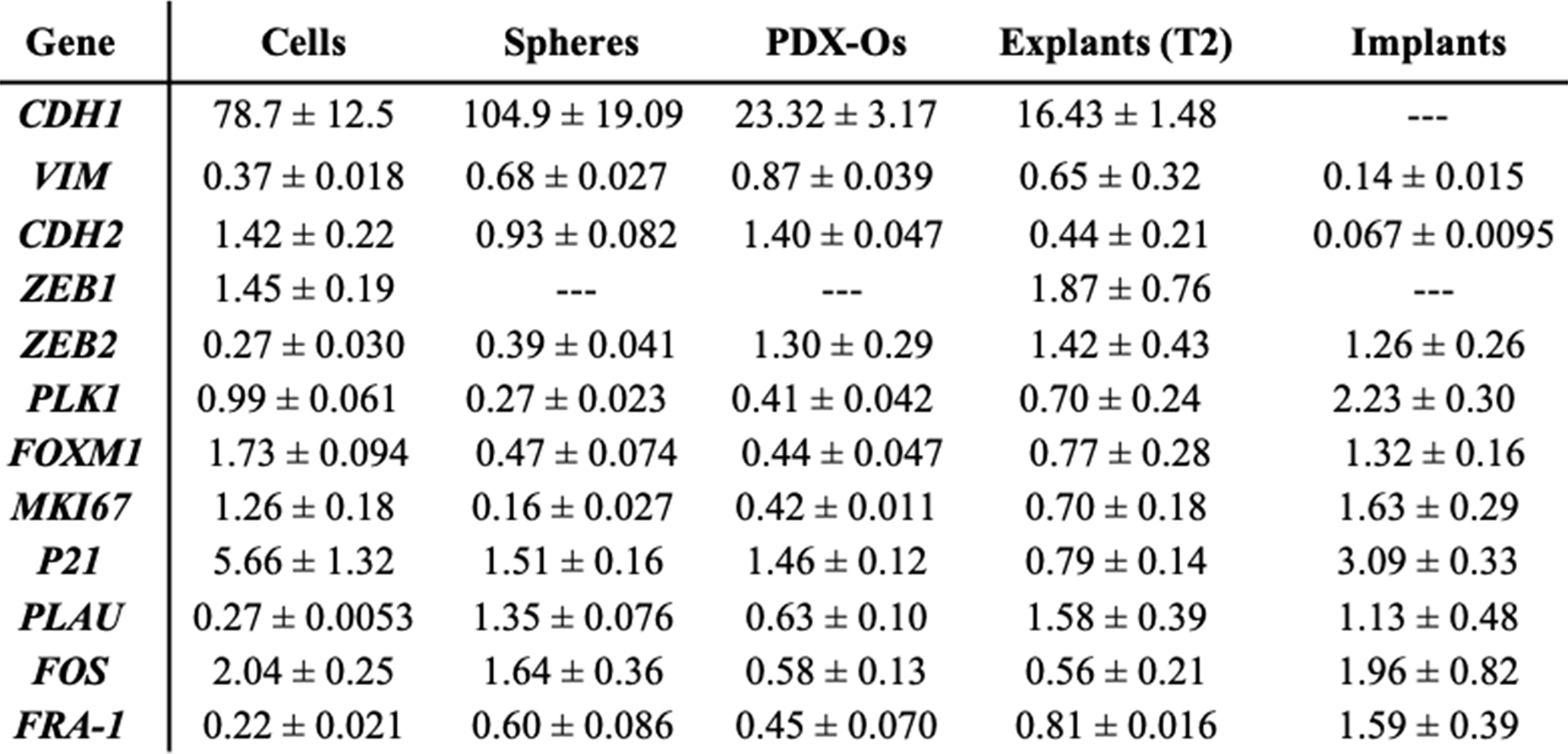

Supplement: S4 Fig — All data is shown as fold change ± SEM normalized to DMSO treatment controls. (TIF) [file pone.0226464.s004.tif]

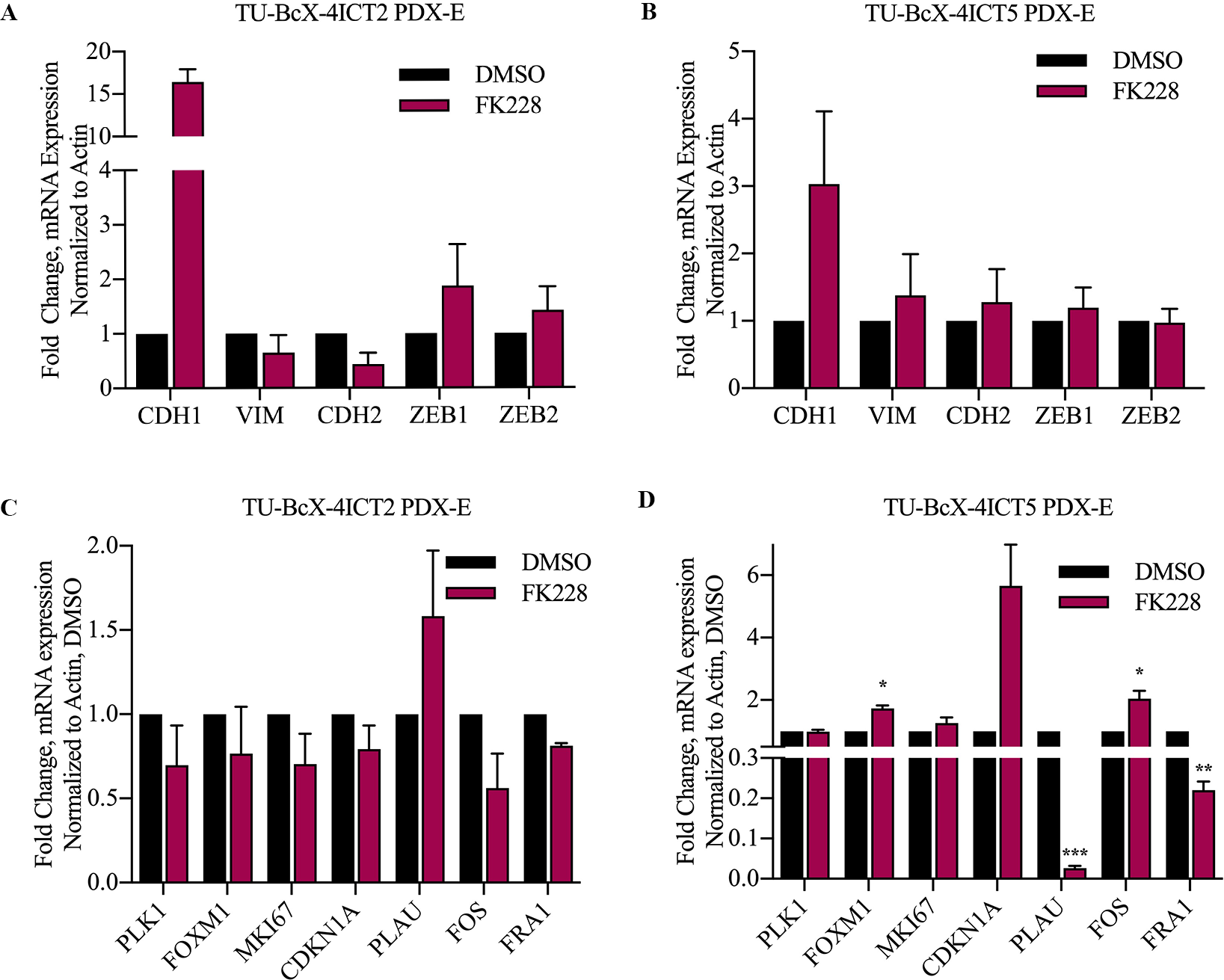

Supplement: S5 Fig — Expression of EMT mRNAs (CDH1, VIM, CDH2, ZEB1, ZEB2) were analyzed using qRT-PCR. Romidepsin was evaluated in TU-BcX-4IC tumor pieces implanted in SCID/Beige mice. Drug effect studies were (A) short term (72 hours) or (B) long term (15 days). qRT-PCR analysis was repeated with genes affected by romidepsin treatment compared to DMSO control based on RNA sequencing analyses (PLK1, FOXM1, MKI67, CDKN1A, PLAU, FOS, FRA-1). Drug effect studies were (C) short term (72 hours) or (D) long term. Black bars represent DMSO; maroon bars represent romidepsin treatment (100 nM, 72 hours). All experiments were run in triplicate. Error bars are shown as S.E.M. (TIF) [file pone.0226464.s005.tif]

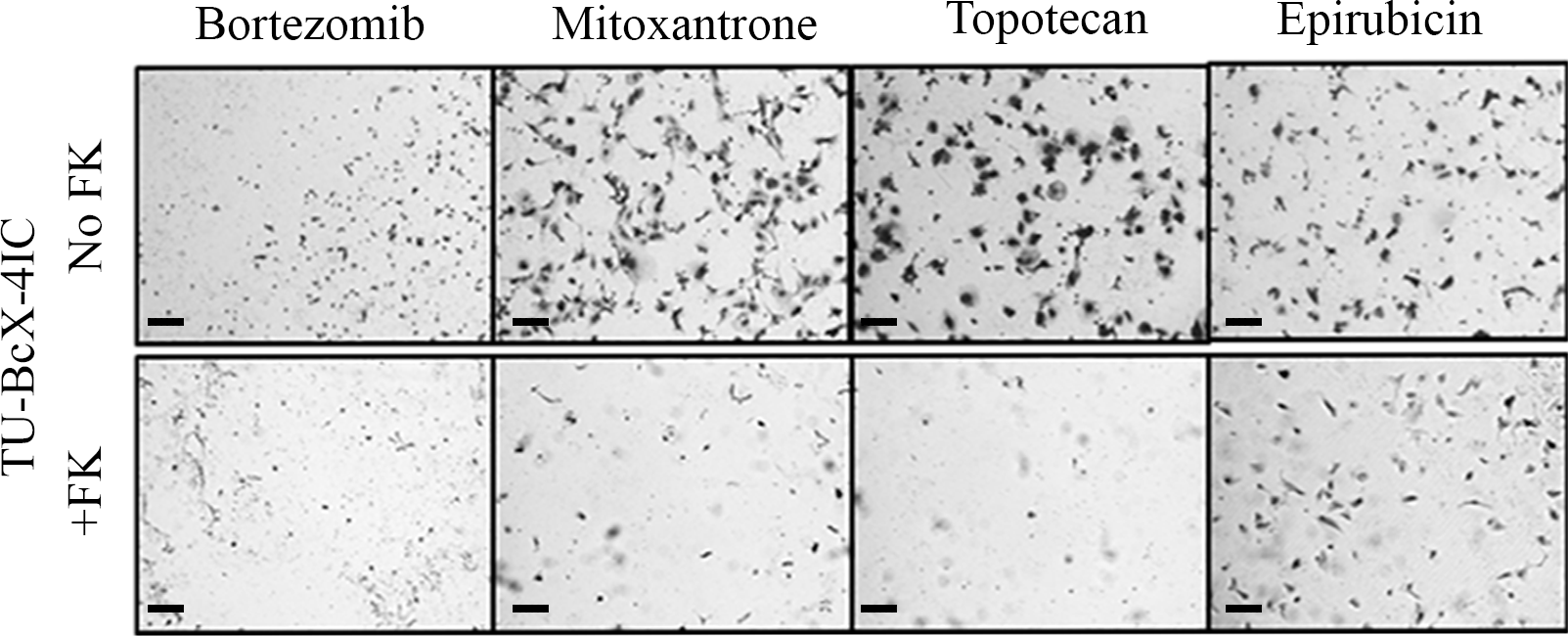

Supplement: S6 Fig — Crystal violet staining of TU-BcX-4IC cells pre-treated with romidepsin for 48 hours (50 nM), or without romidepsin, and then subsequently treated with the NCI oncology drug set. “FK” denotes FK228, or romidepsin, treatment. Scale bars represent 0.25 mm. (TIF) [file pone.0226464.s006.tif]

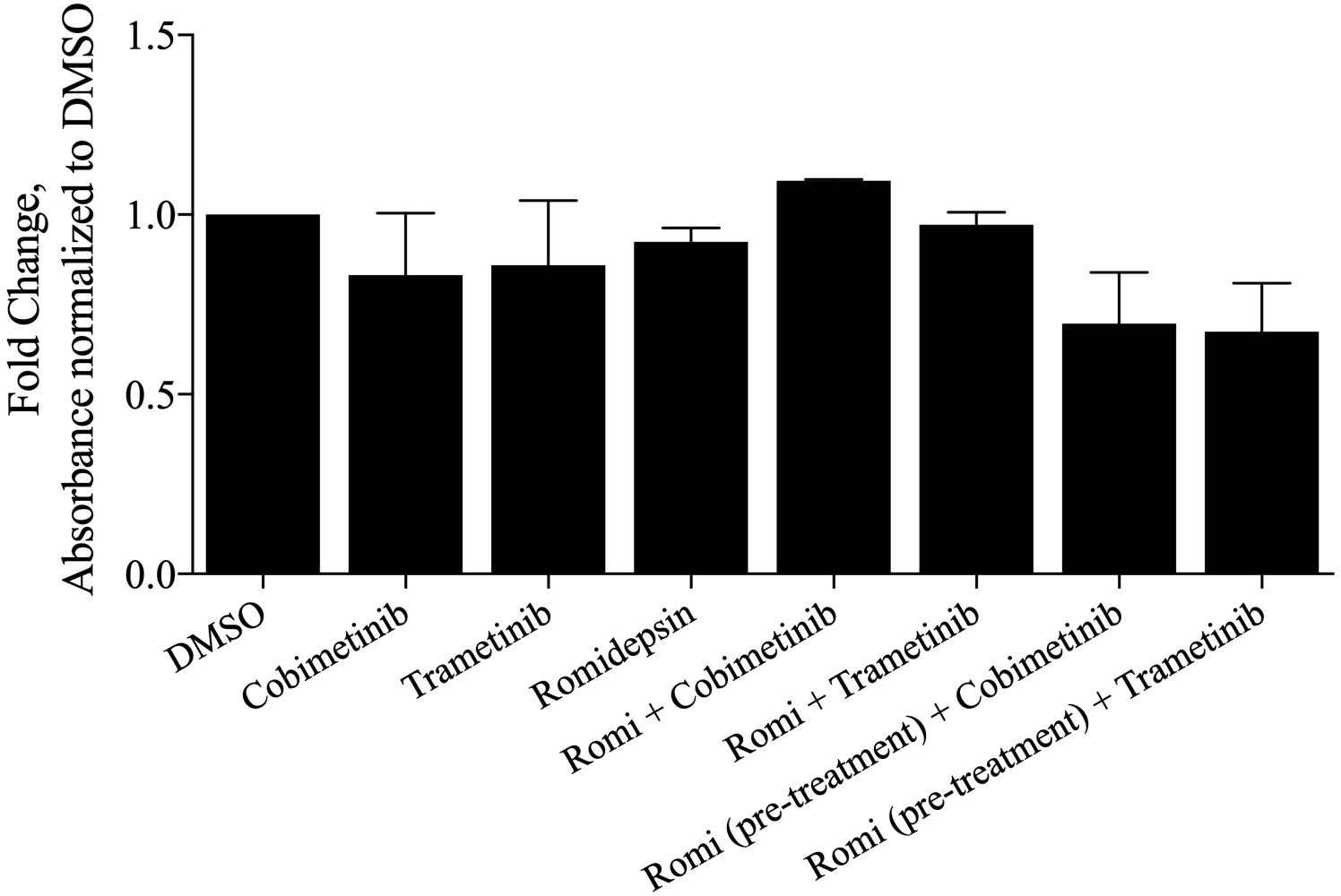

Supplement: S7 Fig — TU-BcX-4IC cells were concomitantly treated with romidepsin and a MEK 1/2 inhibitor. Pre-treatment with romidepsin was studied in TU-BcX-4IC cells by either pre-treating with romidepsin (50 nM) or not pre-treating for 48 hours, and then treating with cobimetinib or trametinib (1 μM). Crystal violet stained cells were lysed and absorbance was measured at 570 nm to quantify staining results. (TIF) [file pone.0226464.s007.tif]
